# Rethinking AI in clinical decision support: a framework for reciprocal human-AI interaction

**DOI:** 10.3389/fdgth.2026.1887161

**Published:** 2026-09-11

**Authors:** Colin John Greengrass

**Affiliations:** School of Medicine, Royal College of Surgeons in Ireland – Medical University of Bahrain, Busaiteen, Bahrain

**Keywords:** artificial intelligence, clinical decision support, deskilling, diagnostic reasoning, human-AI interaction, illness scripts, metacognition, BRACE

## Abstract

Clinical AI systems increasingly match or exceed clinicians on some diagnostic benchmarks. Yet this reveals little about how AI output functions within clinical reasoning or how repeated reliance affects clinicians' independent capability. This paper proposes Bounded Reciprocal Adaptation for Clinician Engagement (BRACE), a framework for AI-enabled clinical decision support centred on the clinician-AI interaction rather than the model output. BRACE links three levels of support. Within a case, it makes uncertainty visible, preserves reasoning state and selectively prompts verification. Across encounters, it supports the development and maintenance of independent reasoning through bounded adaptation and formative review. Institutionally, it separates developmental interaction from evaluative use and routes clinician-initiated reports of AI error or contextual mismatch through governed review. Its governing principle is to provide sufficient support for safe care while preserving the work required for continued independent development. Adaptation is bounded in what the system may infer or modify and how interactional data may be used. These bounds are essential because a reasoning record has developmental value only if clinicians can reason freely, without withholding uncertainty, doubt or provisional judgements out of concern that they may later be used evaluatively. These provisions address design inversion, in which support intended to reduce cognitive vulnerability instead amplifies it: acutely through overreliance and cumulatively through loss or distortion of independent capability. Repeated cognitive and metacognitive offloading to AI may disrupt illness-script formation, maintenance or accuracy, resulting respectively in script-level forms of never-skilling, deskilling or mis-skilling. When AI displaces the discrimination needed to represent an unfamiliar presentation, the corresponding illness script may not form; this risk may be concentrated in ambiguous cases that also attract greater support. The central hypothesis is that the amount and type of cognitive and metacognitive work preserved during a BRACE-mediated encounter should predict independent capability subsequently demonstrated without AI. Evaluation pairs Tier 1 interactional measures derived from ordinary use with Tier 2 external anchors of later AI-off performance. Within-clinician longitudinal analyses test whether preserved cognitive and metacognitive work predicts those outcomes, while inference-drift audits test whether adaptation remains within its declared bounds.

## 1 Introduction

Artificial intelligence has shown rapidly improving performance across diagnostic, predictive and pattern-recognition tasks in healthcare (1, 2). Brodeur et al. (3) report that an advanced large language model outperformed physician baselines across several diagnostic and management-reasoning experiments, including a blinded second-opinion task using emergency-department cases. Yet, randomised studies also indicate that strong model capability does not necessarily improve physician decision-making when deployed as clinical support (4, 5). Thus, seemingly, model capability and clinical benefit are therefore not equivalent.

In this paper, AI-enabled clinical decision support systems (AI-CDSS) refer to tools that use machine learning or generative AI methods to produce, rank, explain or adapt diagnostic and management recommendations. Research on AI-CDSS increasingly recognises barriers related to workflow, trust, task-technology fit and usability. Comparatively less attention has been directed towards how these systems should support the cognitive and metacognitive work of clinical reasoning, including monitoring whether confidence is warranted, reopening a diagnostic frame, maintaining alternatives across interruption, and distinguishing evidential strength from model assertiveness (6–8).

This gap shifts the unit of design and evaluation from the model output alone to the joint clinician-AI system. What that system achieves depends on how outputs are interrogated, corrected and integrated under time pressure, cognitive load and uncertainty. Explainability addresses part of this interactional problem but, as Section 3.2 argues, explanation alone cannot establish whether the clinician can detect, challenge or repair an unsafe recommendation.

The author's earlier work conceptualised AI support for clinical reasoning as cognitive scaffolding within a single encounter. This framework inherits this three-mode account of AI-supported reasoning developed in Greengrass (9), but reverses its system-side profiling of clinician cognitive capacity. Building on that account, Bounded Reciprocal Adaptation for Clinician Engagement (BRACE) extends scaffolding across encounters, asking whether immediate gains preserve capability when AI is unavailable or its output is contested or wrong. Emerging evidence that successful assisted performance may conceal poorer post-withdrawal performance makes that question particularly clinically relevant (10–12).

The effects of AI support on clinician capability may extend beyond the immediate encounter. If AI repeatedly supplies generation, discrimination, synthesis or monitoring that the clinician would otherwise perform, immediate performance may improve. At the same time, opportunities to exercise or develop independent capability may be reduced.

The possible consequences include deskilling of capability already held, never-skilling where relevant capability does not form, and mis-skilling where erroneous or biased output becomes incorporated into later reasoning. Section 8 distinguishes these mechanisms more precisely.

BRACE accordingly operates at two clinical timescales. Within an encounter, it supports diagnostic reasoning by making uncertainty visible, selectively prompting verification and preserving active hypotheses, discriminators and unresolved questions. Across encounters, it supports continuing independent development through case-bounded adaptation, optional formative review and periodic AI-off anchors.

Supporting development across encounters requires a longitudinal record of the support delivered and the cognitive and metacognitive work preserved. Because that record could be repurposed to judge competence, BRACE treats capability preservation and inferential restraint as linked requirements. Three declared boundaries follow. Inferential bounds limit what the system may conclude about the clinician from interactional data. Adaptive bounds limit which aspects of support may change and on what basis. Data-use bounds restrict access to and repurposing of the interactional record. Together they create a clinician-controlled protected developmental space in which interactional data can support learning and bounded adaptation, without becoming a judgement about the person. Separately, clinician reports of erroneous, unsafe or contextually inappropriate AI output can enter governed review rather than directly altering the model or its configuration.

BRACE is not intended as a general framework for every clinical use of AI. Procedural and psychomotor skills, perceptual tasks considered independently of the reasoning they inform, documentation and administrative functions, and patient-facing systems involve different mechanisms and outcomes. Interprofessional and team reasoning also changes the unit of interaction and is largely deferred within the present clinician-AI dyadic account. At the technological level, BRACE is an overarching interaction and governance framework rather than a particular model, interface or product; its provisions must be configured according to intended use, evidence base, operational context and characteristic failure modes.

BRACE is founded on the hypothesis that the amount and type of case-relevant cognitive and metacognitive work preserved during AI-supported encounters predict the clinician's subsequent independent capability without AI. Its design corollary is that the system should provide sufficient support for safe care while preserving the work required for continuing independent development. The amount and form of support may therefore vary with the demands of the current case, system uncertainty and recent interactional patterns in comparable cases, without becoming evidence of a fixed characteristic of the clinician. In this context, case demand refers to the cognitive and metacognitive demands created by the clinical problem itself, including diagnostic difficulty, novelty, atypicality, uncertainty and the amount and complexity of evidence that must be integrated.

The paper proceeds from the cognitive and metacognitive constraints on clinical reasoning in Section 2 to limitations of current AI-CDSS design in Section 3. Sections 4 and 5 develop the reciprocal architecture and its inferential boundaries; Section 6 illustrates the framework in a clinical trajectory; Sections 7 and 8 address calibration, learning and skill risk; Section 9 specifies the protected developmental space; and Section 10 sets out limitations and a programme for empirical evaluation and falsification.

## 2 Cognitive and metacognitive constraints on clinical reasoning

BRACE is derived from established constraints on clinical cognition, learning and metacognitive control. Three general constraints are central. First, limited attentional control and working-memory capacity restrict how many hypotheses can be maintained, compared and revised. When demands exceed that capacity, the problem representation may narrow, premature closure becomes more likely and sensitivity to disconfirming evidence declines (13–15). Second, expertise improves cognitive efficiency without eliminating vulnerability, and overconfidence is not confined to inexperienced clinicians (16, 17). Third, clinical reasoning occurs amid interruption, task switching and handover, which fragment attention and increase memory burden (18–20). These vulnerabilities fluctuate within the same clinician according to case novelty, workload, fatigue and stage of the diagnostic process. Whether and how much support is useful must therefore depend on the reasoning conditions in the particular case rather than on a stable characteristic of the clinician.

These constraints interact with the three modes of clinical reasoning. Pattern recognition is efficient and often appropriate, but is vulnerable to early-cue dominance, atypical presentation, time pressure and diagnostic momentum. Scheme-inductive reasoning depends on traversing relevant schema discriminators and can fail when important branches are omitted. Hypothetico-deductive reasoning requires active generation, comparison, retention and revision of hypotheses and is vulnerable to loss of hypothesis continuity across time, handover and delayed results (21, 22). Support should therefore reflect the reasoning in progress (9, 23). BRACE may prompt a diagnostic pause when pattern recognition occurs under risk conditions, scaffold discriminator checking when schema traversal appears incomplete, or preserve reasoning state when hypotheses must persist over time. The purpose is to support reasoning in the case, not to categorise the clinician.

Effective clinical reasoning depends not only on clinical knowledge and first-order reasoning, but also on metacognition, the monitoring and regulation of one's own thinking as reasoning unfolds. Throughout this paper, cognitive refers to the first-order operations of diagnostic reasoning, including hypothesis generation, schema activation and traversal, evidence integration and discrimination between alternatives. Metacognitive refers to the second-order processes that supervise those operations. Metacognition is conventionally divided into monitoring, the ongoing appraisal of reasoning expressed through confidence, felt uncertainty and recognition that evidence may be insufficient, and control, the regulatory actions that follow, including verification, deferral of judgement, reappraisal and termination of diagnostic search (24, 25). Calibration denotes the correspondence between metacognitive monitoring and actual performance, in this context diagnostic accuracy.

Moment-to-moment metacognitive monitoring differs from broader self-assessment of general ability. Monitoring and control remain vulnerable to cognitive load, fatigue and cumulative workload (18, 19, 26). Diagnostic difficulty may therefore arise not only from missing knowledge or faulty first-order reasoning, but also from failures of metacognitive monitoring and control. Uncertainty may remain unrecognised, or disconfirming evidence may fail to prompt reappraisal or verification.

These are situated vulnerabilities produced partly by the conditions under which reasoning occurs, including the design of the CDSS and other systems with which clinicians work; they should not be treated as fixed deficiencies of the clinician.

The design implication is that AI may indeed support first-order reasoning and metacognitive monitoring, but should not perform the latter on the clinician's behalf. BRACE may make uncertainty, evidential insufficiency and unresolved alternatives visible, enabling verification, deferral or reappraisal. It should not declare the reasoning adequate, because doing so would substitute for the clinician's own monitoring rather than support it.

Earlier formulations of cognitive load theory distinguished three sources of demand (27): intrinsic load, arising from the complexity of the case relative to the clinician's expertise; extraneous load, created by how information and activity are structured rather than by the clinical problem itself; and germane load, associated with processing that supports learning and schema development. Later formulations treat only intrinsic and extraneous load as basic categories, with germane load referring instead to working-memory resources directed towards intrinsic rather than extraneous processing (28). This taxonomic difference does not alter BRACE's claim: whether described as germane load or intrinsic processing, the schema-related operations through which clinical schemas are accessed, assembled and applied must be preserved.

Extraneous load created by duplicated information, unnecessary documentation or poor interface design contributes nothing and can be reduced without loss, whereas the operations that impose germane demand are what build clinical capability. Some demands arise from the working environment rather than from the case or its presentation and are easily mistaken for avoidable burden. Filtering and prioritising information, managing interruptions, reconstructing reasoning state and deciding where to direct attention are skills clinicians must retain in practice. If AI routinely performs these functions, it may make the immediate task easier while potentially reducing the clinician's ability to manage comparable complexity independently. BRACE therefore does not seek to minimise all cognitive load. It reduces burdens that serve no clinical or developmental purpose while preserving or scaffolding the load-management skills, first-order reasoning and metacognitive monitoring and control required for independent practice.

Research on learning also explains why loss of capability may remain hidden. Clinical work is not merely the application of what has already been learned; each encounter is also an occasion on which reasoning is exercised and consolidated, or is not. Performance achieved with assistance therefore does not reliably indicate what has been learned from the encounter, because conditions that make acquisition effortful can also strengthen retention and transfer (29). AI support may supplant precisely those conditions.

These constraints make the clinician–AI interaction, rather than either the clinician or the AI alone, the appropriate focus of analysis. Two interactions may produce the same immediate clinical decision while preserving different amounts and types of cognitive and metacognitive work. Evaluation based on immediate decision performance alone would therefore miss a difference that is central to BRACE. Section 3 examines this limitation in current AI-CDSS design and evaluation, and Section 4.4 develops the interaction trajectory as the relevant unit of analysis.

## 3 Limitations of current AI-supported clinical decision support

### 3.1 The performance-centred paradigm

Measures of output quality, including recommendation accuracy, ranking performance and probabilistic calibration, are necessary for establishing model performance but do not establish how an output functions within clinical reasoning. The effect of a technically identical recommendation may differ according to when it is presented, how it is framed and what cognitive demands the clinician is managing: it may support deliberation, be dismissed without adequate consideration, or precipitate premature closure (30, 31). Evaluation confined to the individual encounter also cannot show how repeated support affects subsequent independent capability. AI-CDSS must therefore be evaluated at two levels: its effect on reasoning within the case and its cumulative effect across encounters, neither of which can be inferred from model performance alone.

### 3.2 The limits of explanation

Explainable AI (xAI) is commonly treated as a bridge between model output and clinician oversight (32). Explanation can be useful when it fits the clinician's reasoning needs, supports the working model of the problem and does not add unnecessary cognitive burden (33–35). A clinically useful explanation should help distinguish alternatives, show which evidence supports or weakens them, and make missing information visible. Without those functions, apparent transparency may leave the clinician no better able to decide whether a recommendation should be accepted, challenged or revised.

Moreover, interpretability and explainability are not equivalent. An interpretable system, such as transparent rule-based logic or an established clinical score, can be examined directly. An explainable but computationally opaque system instead produces a separate account of its output. That account, however, may not faithfully represent the computation it purports to describe (33). The explanation is therefore itself an output that must be evaluated. Reading an interpretable system may constitute part of verification; reading a generated rationale does not remove the need to verify the system that produced it.

The practical consequences of this distinction vary with the opportunity for verification before a decision or action is required. During a time-critical procedure, particularly where an intervention is difficult to reverse, the clinician may have little opportunity to interrogate a generated rationale before acting. An outpatient risk estimate may permit more deliberate review. Even then, the opportunity to review should not be mistaken for evidence that the explanation is reliable: fluency is not evidence of correctness, and a generated rationale cannot by itself establish that the model has been validated for a case like the one at hand.

Zhang et al. (36) propose three properties for managing uncertainty about whether a particular AI output is appropriate: detectability, correctability and non-criticality. Detectability and correctability concern whether an erroneous output can be recognised and then corrected or overridden without disproportionate effort. Within diagnostic reasoning, non-criticality means that an incorrect AI suggestion cannot by itself determine the problem representation, close the differential or trigger consequential action. The suggestion remains provisional, while alternative hypotheses and the evidential requirements for clinical action remain independent of the AI output. The absence of an AI alert must likewise not be treated as evidence that a diagnosis is absent. Explanations may support detectability, but they do not by themselves provide correctability or non-criticality. AI-CDSS therefore requires an interaction architecture that helps clinicians identify and correct problematic outputs while containing the effects of errors that remain unnoticed.

### 3.3 Automation bias, design inversion and metacognitive offloading

Automation bias describes the tendency to accept automated recommendations without sufficient verification or to neglect disconfirming evidence. It is commonly expressed as omission, where a problem is missed because no alert appears, or commission, where incorrect system advice displaces a correct independent judgement (30, 37).

When clinicians trust a system, reliance may extend beyond accepting its recommendations to allowing it to carry part of the underlying reasoning work. In the present context, this is cognitive offloading: the transfer of a first-order operation such as recall, synthesis, detection or ranking to an external aid (38).

Transferring first-order reasoning operations to AI does not remove the need for metacognitive work. Metacognitive evaluation shapes whether those operations are offloaded in the first place (39), while continued monitoring and control are needed after offloading has occurred. The relevant risk within diagnostic reasoning is that reliance on AI may displace these metacognitive operations as well. In metacognitive offloading, the clinician ceases independently to judge whether the underlying reasoning is adequate and whether the AI output is accurate, sufficiently supported and applicable to the case. Within BRACE, the preservation of both first-order cognitive work and second-order metacognitive monitoring and control is therefore a critical design requirement.

This paper proposes the term design inversion to describe an interaction in which AI support intended to mitigate a cognitive vulnerability instead increases it. Design inversion may occur acutely through overreliance or metacognitive offloading, and cumulatively when repeated offloading reduces opportunities to develop or maintain independent capability.

A handover summariser may preserve information while hardening diagnostic momentum if uncertainty, deferred alternatives and reopening conditions disappear. An early diagnostic or triage recommendation may reduce workload while encouraging premature closure if resolution is supplied before alternatives have been discriminated. A confidence display may reassure rather than calibrate if it is detached from missing data, evidential limitations or model instability.

Offloading can improve immediate performance where the aid is accurate, while reducing the planning and monitoring that the unaided task would require. Where an aid reduces the cost of executing a step, anticipatory reasoning confers little advantage, and the task is approached through successive action and correction rather than through prior deliberation. AI support does more than reduce workload; it may change how a task is approached. That modified approach may then persist when the AI is unavailable, even though it may be less effective without that support. For example, a clinician accustomed to stepwise AI prompts may continue to reason incrementally when those prompts are absent, rather than planning ahead (40).

Repeated offloading of first-order reasoning and second-order monitoring may shift the clinician from operator to supervisor, with the system performing part of the reasoning and the clinician checking a result already produced. Bainbridge's (41) irony of automation and the out-of-the-loop performance problem described by Endsley and Kiris (42) capture the resulting difficulty: automation of routine components may erode the skills required to intervene effectively when automation is unavailable or wrong.

This paper proposes the term augmentation paradox for the risk that follows. An AI system may improve the apparent speed, consistency or quality of reasoning within a single encounter precisely by assuming the cognitive and metacognitive work through which the clinician maintains independent understanding, the capacity to detect erroneous output and the capacity to continue safely without the system. The paradox is not an objection to augmentation. It states the condition that safe augmentation must meet: support should improve practice without leaving the clinician less able to explain, challenge, correct or continue safely when the system is unavailable, misleading or operating outside its intended scope.

### 3.4 From transactional to reciprocal interaction

These adverse effects are contingent rather than inevitable. Heudel et al. (43) report automation bias as particularly pronounced in ambiguous or low-prevalence cases, under time pressure, and where interfaces encourage default acceptance without requiring justification or independent verification. Ambiguity and time pressure are features of clinical work and may increase the need for decision support at the same time as they increase the risk of overreliance. Whether an interface invites acceptance or supports independent verification, however, is a design choice and therefore an appropriate target for intervention.

Evidence from Ong et al. (44) reinforces the need for independent verification. In a crossover study of clinicians working challenging ophthalmology cases with and without conversational AI support, confidence rose and self-rated cognitive burden fell across outcome groups, including cases in which a correct answer became incorrect and cases in which an incorrect answer was retained. Overall calibration deteriorated, and unjustified increases in confidence occurred in approximately one third of cases. Reduced effort and increased confidence therefore do not establish that the reasoning displaced by AI was sound.

Conventional AI-CDSS interactions are fundamentally transactional: the system produces an output and the clinician accepts, rejects or modifies it. Such interactions provide little basis for recognising longitudinal patterns such as repeated acceptance without interrogation, failure to revisit deferred alternatives, confidence that does not change after contradiction, or incomplete schema traversal across similar cases. BRACE therefore reframes the design problem around a reciprocal clinician-AI interaction in which the cognitive and metacognitive vulnerabilities of clinical reasoning, and the limits of the system, are made visible and addressed over time. Section 4 develops that reciprocal architecture.

**Figure 1:**
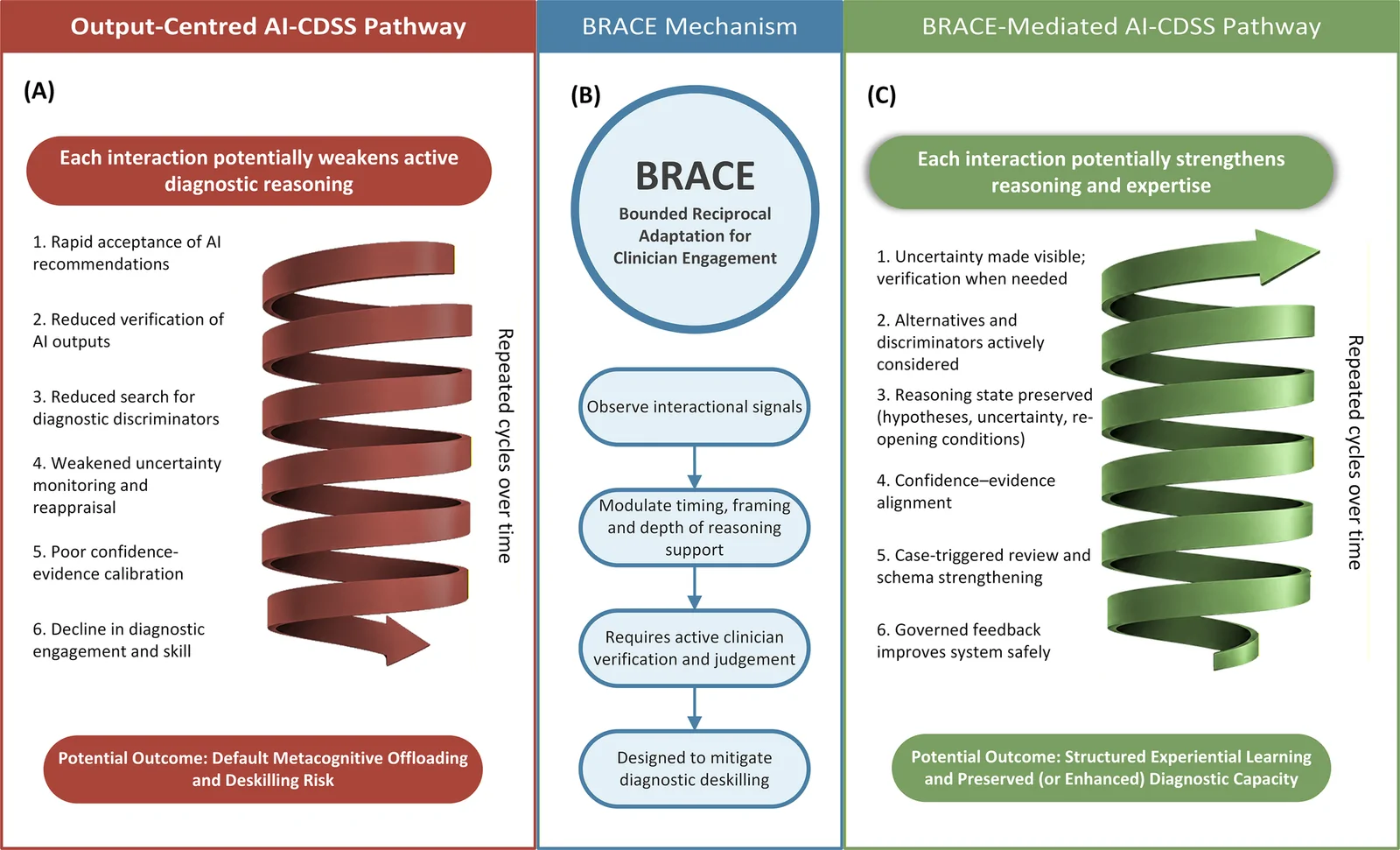
Contrasting AI-CDSS interaction pathways. **(A)** Output-centred AI-CDSS pathway, in which repeated interactions may promote premature acceptance, reduced verification, metacognitive offloading and developmental skill risk. **(B)** BRACE (Bounded Reciprocal Adaptation for Clinician Engagement) mechanism, in which bounded reciprocal adaptation modulates the timing, framing and depth of reasoning support while maintaining clinician-led verification, reappraisal and judgement. **(C)** BRACE-mediated AI-CDSS pathway, in which repeated interactions may support diagnostic reasoning, experiential learning and preservation of independent capability. Spiral arrows represent repeated clinician-AI interactions over time.

Figure 1 contrasts two trajectories arising from repeated clinician-AI interaction. The difference between them does not lie in the accuracy of any single output, but in the mechanism operating between clinician and system over time.

## 4 Reciprocal human-AI interaction

### 4.1 The joint cognitive system as the design unit

Section 2 set out the constraints on clinical reasoning, which arise from limited attentional and working-memory capacity and from the vulnerability of metacognitive monitoring and control. The limits of AI systems arise differently, from the conditions in which they are deployed, from the data on which they are trained, and from the information to which they have no access. Retrieval, calibration and output consistency may deteriorate under contextual pressure in ways that are not immediately visible to users (45); performance may fail to generalise because of dataset shift, shortcut learning or mismatch with the local context (46, 47). Clinically important information may also be unavailable to the system altogether, including findings from physical examination, patient circumstances and preferences, local service conditions and contributions from the wider clinical team.

Neither clinician nor AI can therefore be assumed to provide uniformly reliable supervision of the other. The appropriate design unit is the combined clinician-AI system, or joint cognitive system (48). Within that system, the clinician is not a component to be engineered. What is open to design is the interaction and its trajectory: across repeated encounters the clinician learns how to work with the system, while the system, within declared bounds, learns how to work with that clinician. This adaptation is individualised. It responds to the needs each clinician shows in the current case and in recent comparable cases, and to how each clinician engages with the system, expressed in patterns of reliance, challenge, verification and stated confidence rather than in any inferred attitude or characteristic. This reciprocal adjustment eases the individual clinician into effective interaction and is developed in Sections 4.2 and 4.4. The presence of a human in the loop is not itself sufficient (Section 3.3). Effective oversight depends on how cognitive, metacognitive and computational work are distributed, how the limits of each party are made visible, and how their contributions are coordinated during interaction.

This perspective also changes how failure is interpreted. A problematic interaction may reflect interactional mismatch, a poor fit among the clinician's current reasoning needs, the system's design, the clinical task, the available information and the care environment, any of which may be the source. Research on task-technology fit asks whether a system fits the task and workflow (7). Interactional mismatch concerns whether it fits the reasoning process currently under way. Because interactional data alone cannot distinguish among these possible causes, a failed interaction cannot legitimately be converted into a judgement of clinician competence. Whether reciprocity is safe is therefore a property of interaction design, not simply of clinician competence (Section 4.2).

### 4.2 Reciprocity as a design requirement

In this paper, reciprocity describes a design orientation in which clinician and system adapt to one another over time while retaining asymmetric roles. The clinician remains responsible for clinical judgement: examining the patient, integrating incomplete evidence, communicating uncertainty, incorporating patient values and preferences, and coordinating with the wider team (49). The clinician also remains accountable for action or inaction. The AI-CDSS functions as a cognitive and metacognitive scaffold. It may organise information, make uncertainty visible, prompt verification and preserve reasoning state, but it does not become a co-decision-maker, assessor or proxy supervisor.

The case for retaining clinician judgement is epistemic as well as ethical. On Lange's (50) total-evidence account of AI deference, an AI output contributes evidence to the clinician's judgement rather than replacing the reasons on which that judgement rests. Treating the system instead as an epistemic authority (51) pre-empts the clinician's own reasoning. This is AI pre-emptionism (50): the recommendation substitutes for, rather than contributes to, the generation, discrimination and reappraisal required for independent judgement. BRACE therefore treats AI output as a contribution to clinician reasoning, not as a replacement for it.

BRACE accordingly enters the augment-vs.-replace debate after accepting the case for augmentation. Systems should acknowledge their limits and defer to human judgement where appropriate (52), while the clinician retains overall judgement and the system contributes consistency and precision (44). BRACE adds a longitudinal condition: the clinician must retain the capacity to reason independently when AI is absent or its output is contested or wrong.

Reciprocity also requires each party to model the other, within limits. The clinician must be able to determine what the system can observe, what falls outside its intended scope, and where its evidence or output remains uncertain. The system, in turn, forms a provisional picture of the reasoning in progress, bounded by the inferential limits defined in Section 5.3. This process is akin to mutual theory of mind (53), without attributing a human-like theory of mind to AI systems, which remains contested.

Reciprocity does not therefore mean shared authority. It means that the clinician's use of the system and the system's support for the clinician can become better aligned over time while responsibility for clinical judgement remains with the clinician. Preserving formal human authority is insufficient if repeated interaction progressively erodes the clinician's capability to exercise that authority independently; as that capability diminishes, so too does the practical effectiveness of human oversight.

### 4.3 Positioning BRACE Among neighbouring frameworks

BRACE draws on several traditions in human-AI interaction without being reducible to any of them. Shared mental model theory emphasises overlapping task representations (54), whereas BRACE requires sufficient coordination without assuming that clinician and system represent the case in the same way. Mixed-initiative interaction examines how initiative passes between human and machine (55); BRACE asks when such shifts are safe and which forms of cognitive and metacognitive work they should preserve. Automation-as-team-player accounts emphasise predictability, directability and common ground (56); BRACE adds a longitudinal developmental concern because interaction with AI may affect subsequent independent capability. Adaptive automation commonly responds to inferred operator state (57), whereas BRACE deliberately restricts adaptation to case-bounded, observable interactional signals.

Recent clinical-AI frameworks address related problems at different levels. Ong et al. (58) identify high automation combined with high clinician agency as desirable and propose safeguards including training, minimum unaided practice and interruptions to AI availability. BRACE shares the objective of preserving agency but locates capability protection within the interaction itself, supplemented by clinician-controlled AI-off anchors (Section 9). Li et al. (59) similarly locate the problem at a different level, treating intelligence as a property of the human-AI team and evaluating that team in real workflows through accuracy, safety, workload and patterns of acceptance, editing and override in their TRIAD framework. Its principal level is institutional governance and deployment, whereas BRACE focuses on the reasoning interaction and its consequences across encounters; the two are therefore complementary.

Pan et al. (60) propose a multilevel framework for physician resilience to generative AI organised around cognitive workload shaping, clinical authority governance and organisational safety oversight, including risk-sensitive verification and bounded delegation. BRACE differs in level, outcome and mechanism. Their framework primarily governs the institutional conditions of collaboration; BRACE governs the reasoning interaction. Their outcome is resilience during collaboration; BRACE's developmental outcome is capability retained when AI is absent. Their bounded delegation limits what may be delegated to the system; BRACE's bounded adaptation limits how the system may adapt to the clinician. A governance architecture of the kind Pan et al. propose could therefore contain a BRACE-conformant interaction layer rather than compete with it.

Analogues also exist outside human-AI interaction. Cognitive apprenticeship and entrustment both vary and withdraw support in response to observed activity (61, 62). The analogy ends at assessment: a human supervisor may legitimately use observed performance to judge competence, whereas BRACE may use observed interaction only to configure formative support. Just-in-time adaptive interventions provide a methodological analogue because they alter support in response to changing context (63). BRACE shares this logic of time-varying tailoring but applies it to a safety-critical reasoning interaction while prohibiting person-level inference.

### 4.4 Interaction over time as the unit of analysis

Diagnostic reasoning unfolds across uncertainty, interruption, handover and re-presentation. The relevant unit of analysis is therefore the interaction trajectory, not the isolated decision. This operates at two linked timescales. Within a case, BRACE preserves reasoning state rather than information alone. Across cases, it allows patterns in clinician-AI interaction to influence how support is delivered in subsequent encounters without converting those patterns into a fixed judgement about the clinician.

Within the case, clinical continuity depends on preserving the diagnostic thread: active hypotheses; alternatives deferred rather than excluded and the reasons for deferral; discriminators considered and not yet considered; evidential strength for and against each hypothesis; unresolved uncertainties; pending tests or observations; and conditions that should reopen the diagnostic frame. A shared reasoning workspace of this kind is therefore not simply an interface convenience but a cognitive artefact for preserving hypothesis continuity across interruption, delayed results, handover and re-presentation.

Across encounters, BRACE does not assign a clinician a fixed support category. Clinical expertise is uneven within individuals as well as between them because illness scripts develop through the presentations actually encountered. The same clinician may therefore require little support in one case type and substantially more in another. For BRACE, a case type is a clinically meaningful class of presentation defined by its diagnostic structure and relevant discriminators, rather than by the clinician's performance or the eventual outcome. Low, moderate and high support are conceptual support configurations rather than validated thresholds; their operational criteria require empirical calibration within the clinical setting. They may vary according to case novelty, evidence quality, system uncertainty, stage of reasoning and recent interactional patterns in comparable cases.

Adaptation applies to the delivery of support, not to the underlying clinical evidence. The differential, evidence and declared system uncertainty do not change because a different support configuration is in force. What may change is when the system's resolution is disclosed, whether the clinician is first asked to commit to alternatives or discriminators, whether unconsidered discriminators are surfaced, and whether verification is prompted. A previous coverage gap in a case type, meaning something the case appeared to require but which was not considered (Section 5.1), therefore does not justify different clinical advice on the next similar case; it may justify changing how and when the same evidence and advice are presented.

One boundary is categorical. No developmental or capability-preservation objective may delay the disclosure of information where delay could materially increase immediate risk to the patient. Immediate disclosure is required where the presentation is time-critical, as in suspected stroke within the thrombolysis window; where the clinical state is unstable, as in progressive hypotension or a falling conscious level; or where a critical finding has not yet been recognised by the clinician, as in severe hyperkalaemia on routine bloods. In any of these circumstances the developmental sequence is suspended for that case. The clinician may also request immediate disclosure at any time, with the same effect. This safety override takes priority over every other provision of BRACE. It is triggered by the case, not by a judgement about the clinician, and its activation therefore generates no clinician-level inference. A system that preserved cognitive and metacognitive work at the cost of immediate safety would produce the very design inversion BRACE is intended to prevent.

BRACE distinguishes three levels of adaptation. Case-level adaptation responds only to the current presentation and interaction. Case-type adaptation may use recent within-clinician interactional patterns for the same case type, interpreted against case difficulty, evidence quality and system uncertainty. Such adaptation may be clinician-specific but must remain time-limited, inspectable, challengeable, suspendable and resettable by the clinician. Global adaptation, in which a support configuration follows the clinician across unrelated case types, is prohibited because it would amount to a standing claim about that clinician's capability. Section 5.3 specifies the corresponding inferential boundary.

**Figure 2:**
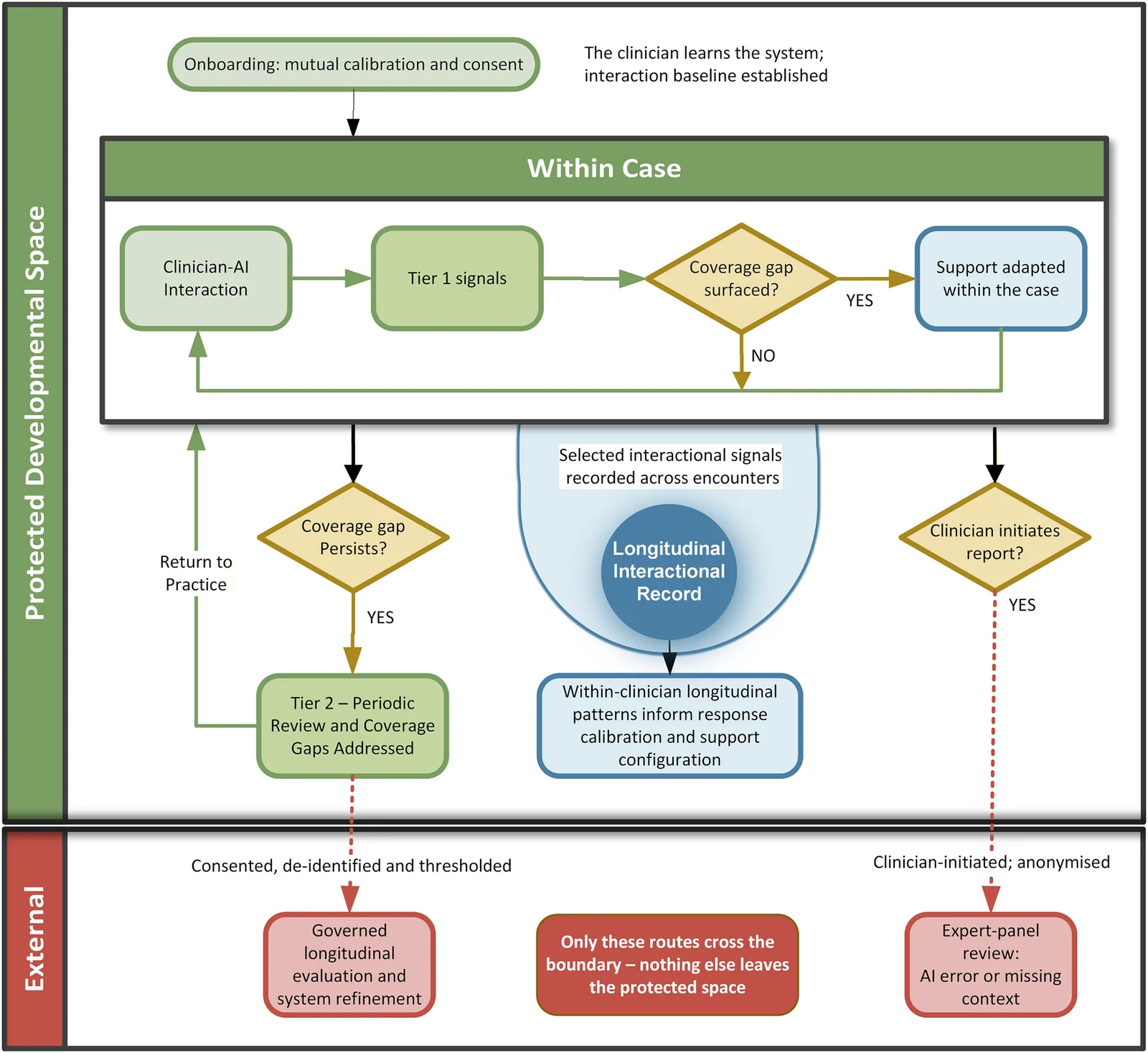
BRACE (bounded reciprocal adaptation for clinician engagement) architecture and protected developmental boundary. Onboarding establishes mutual calibration and consent; within-case interaction generates Tier 1 interactional signals and bounded support, while persistent coverage gaps may prompt optional Tier 2 external-anchor review before return to practice. Selected interactional signals recorded in the longitudinal interactional record inform case-type calibration and support configuration. Only two governed routes cross the boundary: consented, de-identified evaluation and system-refinement data reported only in sufficiently aggregated form, and clinician-initiated anonymised expert review of possible AI error or missing context.

Figure 2 integrates these elements, showing how case-level interaction, longitudinal adaptation and governed data flows are separated by the protected developmental boundary, and where the two governed routes cross it.

## 5 What the system may observe, and what it may not infer

BRACE translates the cognitive and metacognitive principles developed above into observable interactional signals and bounded adaptive responses. The critical distinction is between observing what is happening in a clinical interaction and making a judgement about the clinician. An observation may justify changing the support offered in a case or case type; it does not, by itself, justify an inference about why that support is needed or what the observation says about the clinician more generally.

Each adaptive action requires a permitted mechanism and adaptation level (Section 4.4), and each permitted mechanism rests on an established cognitive or educational basis. Reasoning-state preservation relieves working memory of hypotheses it would otherwise hold against interruption and decay (20, 64). Verification checkpoints operationalise cognitive forcing against premature closure, a major cognitive contributor to diagnostic error (13). Evidence-sufficiency prompting supports metacognitive monitoring before commitment (65). Discriminator surfacing supports scheme-inductive schema traversal (66), and formative review draws on retrieval practice (67).

### 5.1 Observable interactional signals

BRACE restricts adaptation to five classes of observable, case-bounded interactional signal: expressed confidence and its revision as evidence changes; engagement with AI output, including acceptance, challenge, override and verification; diagnostic-pathway activity, including alternatives and discriminators articulated, examined or deferred; temporal features, including the timing of convergence and revision; and coverage of the case representation, including unresolved questions and missing information.

A coverage gap is the absence of something the case appears to require: for example, a relevant discriminator has not been considered, an important alternative has not been generated, or the current representation does not accommodate the presentation. Coverage gaps are what the signals are examined for and what adapted support is intended to address. They are observations about the current interaction, not judgements about the clinician.

No interactional signal is interpretable in isolation. Except where an explicit request or predefined safety condition requires immediate support, adaptation should depend on a convergent pattern interpreted against case difficulty, evidence quality, system uncertainty and stage of reasoning. The signals may therefore indicate what support could be useful without establishing why it is needed. This distinction underpins the inferential boundary developed in Section 5.3.

Where clinically appropriate, some signals can be obtained by eliciting a provisional reasoning state before the system displays its own differential or recommendation. The clinician may be asked to record provisional alternatives, identify important discriminators and indicate confidence in the leading possibilities. This intervention deliberately shapes the reasoning it subsequently observes, but it does so to preserve first-order generation and discrimination together with second-order confidence monitoring. Elicitation is an invitation rather than a gate: the clinician is asked first, and where no provisional reasoning is offered, the system proceeds to its own differential without further delay, and the absence of a response carries no inference beyond the case. Of the signals elicited, confidence receives particular handling: it is recorded as a judgement about the current case and tracked as evidence changes.

Interactional signals may provide tentative cues to the mode of reasoning in use, but are not definitive and require empirical validation. Rapid early convergence on a single diagnosis with little discriminator engagement may suggest pattern recognition, particularly where confidence is high from the outset (21). Incomplete traversal of relevant discriminators may suggest scheme-inductive reasoning (66). Extended revision as sequential evidence accumulates, together with explicit uncertainty and delayed confidence resolution, may suggest hypothetico-deductive reasoning (22). These interpretations must remain conditioned on the case and its stage. Early convergence before relevant evidence is available does not mean the same thing as convergence after sequential evidence has been integrated.

Pattern recognition is less reliable with atypical presentation or discordant evidence (13, 68), so discriminator-based or hypothesis-comparison support may be offered more readily. The system may surface an unconsidered discriminator or plausible alternative where convergence is early or schema traversal appears incomplete, supporting comparison without selecting the diagnosis or replacing clinician ordering.

### 5.2 System-side uncertainty and operating limits

Automated aircraft-landing systems illustrate the value of a defined operating boundary (58): they perform a narrowly specified task and are authorised only under specified aircraft, runway and environmental conditions. Task-specific clinical AI may similarly have clearly defined intended-use limits. General-purpose generative clinical AI is more difficult to bound because it encounters heterogeneous and evolving cases in which relevant information may be missing, presentations may be atypical and clinically important context may be unavailable to the model (58).

The limit of the aviation analogy is instructive. A landing has relatively determinate success criteria and a specifiable input space; an undifferentiated diagnostic encounter often does not, because the clinical problem itself is progressively constructed during the encounter. Displaying uncertainty does not solve this problem. Low confidence indicates uncertainty in an output but does not by itself establish whether the system is appropriate for the case.

The distinction matters most in the cases where support is most wanted. Ong et al. (44) found that AI collaboration improved performance in some cases but conferred no significant benefit on the most difficult cases, where accepting an incorrect AI suggestion was the most common failure. Where a dependable operating boundary cannot be specified, the system should state the relevant limitation, identify the verification its output requires and, where appropriate, decline to provide a recommendation.

### 5.3 Case-directed adaptation and inferential limits

BRACE permits adaptation to case-bounded features of reasoning, such as unresolved uncertainty or incomplete discrimination, but it does not permit those observations to become a characterisation of the clinician. Interactional data must not be used to generate, score, retain or communicate judgements about competence, expertise, intelligence, motivation, diligence, professionalism or trustworthiness; psychological or affective states such as stress, fatigue, burnout or emotional exhaustion; or any enduring personal or professional characteristic.

Case-type adaptation nevertheless requires some use of recent interactional history. The key distinction is between using that information to adjust support and using it to make a judgement about the clinician. Such information may determine a reversible support action within a defined case type, but its relevance is temporary and is superseded as subsequent interaction changes the basis for support.

An architecture implemented according to BRACE must prevent four uses of this information. This information must not be converted into an evaluative judgement about the clinician, compared between clinicians, exported beyond the case type that generated it, or used to inform consequential decisions such as supervision, credentialing or employment. The distinction is architectural rather than merely procedural: this information must be represented only in the form needed to configure the bounded support action, and the two governed routes across the protected developmental boundary carry no version of it. The relationship is analogous to a thermostat reading: it adjusts the heating and is then superseded, and it is not retained as a description of the household.

Within these limits, the system may act as a constrained critical friend, using the interactional interventions described above to support verification, discrimination and reappraisal without acquiring evaluative authority over the clinician (69, 70). This restriction follows from the interactional mismatch described in Section 4.1: because a problematic interaction may have several possible causes, interactional data alone cannot justify a judgement about the clinician.

A known contextual fact may nevertheless justify a limited change in support without becoming an inference about the clinician. For example, a predefined shift duration may trigger a private, optional offer of additional verification prompts without implying that the clinician is fatigued. The trigger must be a predefined institutional fact rather than an inferred state; no explanation for accepting or declining may be requested or recorded; neither response enters the longitudinal interactional record; and support may increase but not fall below the contextual baseline. Supplementary Materials S5, S6 specify the fuller configuration.

### 5.4 Ambient monitoring

BRACE excludes ambient sensing of the clinician. Facial, vocal, physiological or behavioural surveillance would convert a framework based on deliberate interaction into one that infers internal state. Ambient documentation systems involve a different data source and governance problem and are therefore considered separately in Supplementary Material S7.

Table 1 integrates these principles by mapping common AI-CDSS functions from design-inversion risk to bounded BRACE response, the clinician work preserved, and the relevant boundary condition. These are design principles rather than fixed implementation rules.

**Table 1:** Operationalising BRACE: design-inversion risks and bounded interactional safeguards. Each row maps a common AI-CDSS function from the design-inversion risk it carries to the bounded BRACE response, the clinician work that response preserves, and the governance condition limiting that response.

AI-CDSS function	Design-inversion risk	Bounded BRACE response	Preserved clinician work	Boundary condition
Handover or re-presentation support	A summary presents the working diagnosis as settled, making deferred alternatives and uncertainty invisible.	Preserve the reasoning state: active hypotheses, deferred alternatives, evidential gaps, discriminators not yet considered, and reopening conditions.	Hypothesis continuity, reappraisal, handover reasoning.	Only case-bounded reasoning state is recorded.
Early diagnostic recommendation	The highest-ranked AI diagnosis anchors the clinician and accelerates premature closure.	Use event-triggered verification when confidence is high despite incomplete or discordant evidence, or when system uncertainty persists.	Independent verification, alternative generation, diagnostic pause.	Prompts must be selective and low-burden, not routine interruption.
Confidence display	Numerical confidence is treated as reassurance, reducing scrutiny as displayed confidence rises.	Pair confidence with evidence-sufficiency prompts and missing-data visibility.	Confidence–evidence concordance and uncertainty monitoring.	Confidence remains case-specific and is not aggregated into a capability score.
Differential diagnosis list	Clinician attends only to the top-ranked diagnosis; lower-ranked discriminating alternatives are ignored.	Surface unconsidered discriminators and plausible alternatives when convergence is early or schema traversal is incomplete.	Diagnostic discrimination, schema traversal, comparison between alternatives.	The system supports comparison but does not replace clinician ordering or judgement.
Automated synthesis of complex data	AI provides a resolved synthesis, displacing the clinician's work of integrating evidence.	Display unresolved contradictions, evidential gaps, and uncertainty rather than only a completed interpretation.	Evidential integration, sense-making, active judgement.	Support should reduce extraneous load while preserving essential reasoning work.
AI explanation and rationale	Explanation is consumed as a complete answer; uncertainty monitoring and reappraisal are delegated to the system.	Prompt for selected confidence, evidence-sufficiency or reappraisal judgements at risk points.	Metacognitive monitoring, reappraisal, contingency preparedness.	Prompts are triggered by case or system conditions, not inferred clinician states.

## 6 A worked clinical scenario

The following composite scenario traces the design principles through a first presentation, re-presentation and retrospective review. It illustrates recognised diagnostic-safety problems in emergency presentations of pleuritic chest pain; it is not a reported case.

### 6.1 First presentation, 02:00

A 58-year-old patient presents to the emergency department with pleuritic chest pain and mild dyspnoea after a long-haul flight. The clinician is in the eighth hour of a busy shift. Their initial differential includes pulmonary embolism, musculoskeletal chest wall pain, pericarditis and atypical respiratory infection. Reproducible chest wall tenderness makes a musculoskeletal cause appear more likely, and pulmonary embolism is initially considered less probable. The AI-CDSS identifies pulmonary embolism as the highest-risk diagnosis requiring exclusion, while also retaining pericarditis and atypical infection as active alternatives because the available evidence is incomplete and partly discordant.

The encounter is configured as high support because the presentation is atypical, the evidence is discordant and system uncertainty remains high. Before displaying its own differential, the system asks the clinician to record provisional alternatives, the discriminators considered most important and confidence in the leading possibility. This preserves an independent provisional reasoning state before the AI supplies its own resolution. The system then makes its uncertainty visible and uses a selective diagnostic pause rather than a generic alert, asking what finding would most change the working diagnosis.

The reasoning state is preserved as the encounter develops: active hypotheses, missing evidence, deferred alternatives and the conditions that should reopen the diagnostic frame remain visible rather than being replaced by the final working diagnosis. The interactional record also captures the cognitive and metacognitive work preserved during the encounter, including alternatives generated, discriminators examined and verification undertaken. Because the clinician has passed a predefined shift-duration threshold, the system privately offers additional verification support; accepting or declining that offer requires no explanation and neither response enters the longitudinal interactional record. If subsequent disagreement, uncertainty or re-presentation creates a useful learning contrast, the encounter may later be offered for formative review (Section 7.2).

### 6.2 Re-presentation

The patient returns 38 h later. The earlier CT pulmonary angiogram was negative, symptoms have worsened and two handovers have occurred. BRACE carries forward the earlier diagnostic reasoning, not merely the clinical data. The incoming clinician can therefore see that musculoskeletal chest wall pain became the discharge diagnosis, that pulmonary embolism was excluded on imaging, and that pericarditis and atypical infection were deferred rather than excluded.

Because the presentation has changed while previously unresolved alternatives remain open, the encounter again meets the conditions for high support. The system surfaces discriminators relevant to those alternatives rather than simply repeating the previous differential. On re-examination prompted by those discriminators, a pericardial rub is present, the electrocardiogram shows widespread saddle-shaped ST elevation, and echocardiography demonstrates a small pericardial effusion. Acute pericarditis is diagnosed.

The clinically important feature of the example is not that BRACE finds the diagnosis. The diagnosis is resolved through new clinical evidence. BRACE's contribution is that the earlier reasoning state survives the intervening handovers, allowing an alternative previously held open to be reconsidered when the evidence changes. The preserved record supports reappraisal rather than retrospective criticism of the earlier clinicians.

### 6.3 Retrospective review

One week later, the original clinician is privately offered an optional formative review because the encounter combined high system uncertainty, initial clinician-AI disagreement and subsequent diagnostic clarification. Declining the review is not recorded.

The review reconstructs the preserved reasoning trace: the hypotheses considered; discriminators used, omitted or deferred; confidence recorded before the AI differential was displayed and how it changed as evidence emerged; evidence-sufficiency judgements; and the eventual diagnostic outcome. It shows where the working diagnosis was generated, maintained, closed and later reopened. It also identifies the cognitive and metacognitive work preserved during the original encounter, because BRACE hypothesises that this, rather than the level of support itself, should predict subsequent independent capability (Section 10.2).

Where the review identifies a useful learning contrast, the clinician may be offered a Tier 2 anchor, for example an uncertainty-rich vignette involving a similar presentation, completed without AI support to rehearse likelihood revision, discrimination and confidence calibration. The clinician may suspend the review, challenge the system's interpretation, or add contextual information unavailable to the AI or electronic record, including bedside observations, patient preferences and local constraints.

The purpose of the review is reciprocal calibration and learning, not retrospective defence or assessment. Its contents remain within the protected developmental space. If the clinician believes the AI output was unsafe, erroneous or contextually inappropriate, that concern follows the separate clinician-initiated governed review pathway described in Section 9.

## 7 Baseline calibration and formative remediation

### 7.1 Baseline calibration

Early human-automation interaction can shape subsequent reliance, disuse and misuse because users form expectations about system reliability during initial exposure (31, 71). BRACE therefore treats onboarding as baseline calibration rather than technical induction. Calibration introduces clinicians to when and why the system may alter the timing, framing or depth of support, its declared operational limits, and their ability to inspect, challenge, suspend or reset those changes. It should occur in the clinical context in which the AI-CDSS will actually be used rather than through generic software instruction (72, 73).

Calibration proceeds through two passes over uncertainty-rich Script Concordance Testing (SCT)-style cases, in which evidence is disclosed sequentially and the clinician revises likelihood, direction and confidence as the case evolves (74, 75). The clinician first works a set of cases without AI support. This rehearses independent generation, discrimination and confidence revision and establishes an AI-off anchor against which later independent capability can be compared. A comparable set is then worked with BRACE available so that the clinician experiences directly what support is offered, when it appears, and what cognitive and metacognitive work remains theirs to perform.

The contrast between the two passes is itself instructional. Cases should include accurate AI outputs, misleading outputs and outputs generated under the contextual limitations described in Section 4.1. The purpose is for clinicians to experience where the system's apparent fluency may exceed what its available information can justify, and therefore when an output should be verified, qualified or set aside.

Baseline calibration should also establish the boundaries of participation. Clinicians should understand the distinction between routine case-level interaction, optional formative review and separate consent for predefined de-identified measures to contribute to longitudinal evaluation. Access, withdrawal and data-use controls are specified in Section 9.

### 7.2 Formative remediation and BRACE-augmented experiential learning

Selected encounters may be revisited through optional formative review. In this context, remediation denotes formative strengthening of reasoning opportunities rather than a competence determination. One useful structure is the SCT-derived sequential-disclosure format introduced during baseline calibration (Section 7.1), reused here to organise review of evolving diagnostic likelihood, management direction and confidence (74, 75). BRACE uses this structure formatively rather than as a scored assessment. Confidence judgements made across the evolving case can also provide Tier 2 anchor measures for use by the clinician within the protected developmental space. AI may support the generation and variation of SCT-style cases at scale (76, 77).

The content of review is structured by the mode of reasoning in use. Where rapid convergence proved unsafe, review may rehearse the discriminators that would have distinguished the presentation from the pattern it resembled. Where scheme-inductive reasoning showed incomplete traversal, review may make expected discriminators, untraversed branches and unsafe closure points visible. Where hypothetico-deductive reasoning was required, a reasoning tree may show which hypotheses were generated, retained, rejected or reopened, supporting reflection on reasoning continuity. These activities are grounded in deliberate reflection and illness-script development (22, 78), although whether BRACE-mediated review improves subsequent reasoning remains an empirical question.

A further target for formative review is causal understanding. Biomedical knowledge can support diagnostic reasoning by providing causal coherence between pathophysiological processes, clinical findings and diagnostic possibilities, particularly when cases are difficult or unfamiliar (79, 80). Therapeutic reasoning likewise requires clinicians to connect pharmacological knowledge with rational treatment decisions (81). Such understanding may become increasingly important in AI-supported care, where clinicians must be able to recognise recommendations that are superficially plausible but inconsistent with the underlying causal relationships in the case. BRACE-mediated remediation can therefore revisit not only diagnostic discriminators but also the causal links between pathophysiology, clinical findings and therapeutic action.

BRACE thereby extends experiential learning by allowing selected clinical encounters to become structured learning objects. Retrospective reflection is ordinarily constrained by what clinicians can reconstruct of their own reasoning after the event. The preserved reasoning trace provides a contemporaneous record of uncertainty, AI disagreement, discriminators considered or omitted, changes in confidence and points at which a diagnostic frame was maintained or reopened. The purpose is not to judge the clinician retrospectively, but to make the reasoning process available for learning.

Review may extend beyond an individual encounter. Clinicians may revisit their own interactional record to examine patterns across related cases, allowing experience to be compared with an organised representation of the relevant presentation rather than recalled in isolation (22, 82). Script-concordance cases are one vehicle for doing so, but the mechanism does not depend on SCT: the clinical encounter is the learning object, the preserved reasoning trace supplies evidence of the reasoning process, and the relevant schema provides the scaffold through which that experience is interpreted.

This developmental function matters because repeated interaction may shape not only performance in the supported encounter, but which diagnostic capabilities are subsequently available without support. Section 8 examines the corresponding risks of deskilling, never-skilling and mis-skilling.

## 8 Skill risk and metacognitive alignment

### 8.1 Deskilling, never-skilling and mis-skilling

Repeated AI-supported interaction creates at least three distinct risks to independent capability, introduced together by Berzin and Topol (83). Deskilling is erosion of a capability already held, also described as expertise atrophy (50) or loss of expertise (43). Never-skilling is failure to develop a capability because AI displaces the cognitive work through which it would otherwise form, elaborated for medical education by Ke et al. (84). Mis-skilling is acquisition or reinforcement of a distorted competence through uncritical incorporation of erroneous or biased AI output, evident where clinicians abandon correct initial judgements after receiving erroneous AI advice (43). Mis-skilling can therefore arise at any career stage whenever erroneous output is incorporated without adequate verification, rather than only during training.

Ke et al. (84) locate never-skilling principally in formative training, drawing on the expertise reversal effect (85): guidance that supports a learner who lacks an appropriate schema may become redundant or disruptive once that schema has developed. The important qualification for clinical reasoning is that expertise is not uniform across a clinician's practice. A clinician may possess extensive general experience while encountering a particular presentation for which the relevant illness script is weak or absent. In that domain, the clinician again occupies a novice-like position regardless of seniority.

Illness-script development continues beyond certification because new and atypical presentations continue to be encountered throughout a career (22). Never-skilling can therefore occur at the level of a particular clinical representation rather than only at the level of general professional development. This paper proposes the term script-level never-skilling for failure to form an illness script because AI supplied the resolution before the clinician performed the generation and discrimination through which that script would otherwise have developed.

The risk may be concentrated rather than evenly distributed. Clinicians may seek greatest support for unfamiliar, atypical, ambiguous or high-stakes presentations, which are also encounters with substantial script-forming value. An output-centred system may therefore displace independent discrimination precisely where that discrimination would contribute most to development. The relevant variable is not whether assistance comes from AI, a colleague or another resource, but whether the timing and form of support preserve an attempt at retrieval and discrimination before resolution is supplied (86). This selection effect remains a proposition requiring prospective testing.

Never-skilling and script-level deskilling arise through different processes. In never-skilling, the relevant illness script does not adequately form because the cognitive work through which it would normally develop has been displaced. This paper likewise proposes the term script-level deskilling for the case in which the script was previously formed, but access to it becomes progressively weaker through disuse. At severe levels, these two states may look the same on a single unaided assessment because, in both cases, the clinician may be unable to retrieve or use the relevant script. Apparently forgotten material can nevertheless show savings on relearning even when recall or recognition fails (87, 88). On that basis, BRACE predicts that a deskilled script should recover or relearn more rapidly after re-exposure, whereas a script that never adequately formed should show no equivalent savings. Current performance alone may therefore be insufficient to distinguish deskilling from never-skilling.

Memory research suggests that disuse commonly weakens retrieval more readily than storage, with substantial recovery following re-exposure (29, 89). Loss should therefore appear first as reduced differentiation among neighbouring clinical representations rather than disappearance of knowledge altogether. Contrastive exposure is particularly important for maintaining discrimination between similar categories (90, 91), while reconstruction tends to drift towards prototypical representations (92). BRACE therefore predicts that script-level deskilling will appear as increasing confusion among similar presentations, often biased towards the more familiar or prototypical script.

These propositions generate the central directional prediction of BRACE: within the same clinician, the amount and type of cognitive and metacognitive work preserved during an AI-supported encounter should predict independent capability subsequently demonstrated without AI. Support level itself is not the hypothesised mechanism; what matters is the reasoning work preserved under that support. Section 10.2 specifies the conditioning required to test this relationship.

Bastani et al. (11) provide an analogue from formal instruction: unrestricted AI improved assisted practice but was followed by poorer performance when assistance was removed. This demonstrates that supported performance can conceal a deficit that becomes apparent only under unaided conditions. Clinical practice differs substantially from formal instruction: learning is incidental, cases are less controlled and consequences are clinical rather than educational. Whether the same effect occurs with AI-CDSS has not been established. BRACE treats it as a testable possibility rather than an assumed fact.

Existing proposals often locate capability protection outside the AI-supported encounter. Abdulnour et al. (93) emphasise post-case supervisory discussion; Ke et al. (84) emphasise establishing capability before AI is introduced; and Ong et al. (58) propose periods of unaided practice. BRACE can incorporate analogous functions through post-case review and AI-off anchors, but adds a distinct requirement: some protection must occur during the encounter itself. The developmental opportunity exists at the point where the clinician either performs the relevant discrimination or receives the resolution before doing so. Eliciting provisional alternatives and discriminators before AI disclosure is intended to preserve that opportunity.

### 8.2 Metacognitive alignment

A clinician's principal protection against a wrong diagnosis is the capacity to recognise that it may be wrong. Metacognitive alignment denotes that capacity as it operates during AI-supported reasoning: the ability to detect that a diagnosis is discordant with the available evidence, that the differential remains incomplete, or that the system's output may itself be erroneous. It applies whether the judgement under examination originates with the clinician or the AI. Metacognitive alignment is therefore primarily state- and case-dependent, rather than a stable trait of the clinician.

Alignment is not agreement with AI. Disagreement with a well-supported AI output may reflect accurate detection of a genuine evidential conflict; agreement reached without interrogation may occur even when detection has not taken place. What matters is whether confidence responds to evidence and whether recognised uncertainty leads to appropriate regulatory action such as verification or reappraisal. Detection is useful only if the interface also permits meaningful challenge, but that is an architectural requirement rather than a metacognitive property of the clinician.

Metacognitive alignment is related to, but not captured by, existing measures. Calibration and metacognitive sensitivity describe how confidence corresponds to accuracy but do not reveal the evidential basis of that confidence (94). The same confidence may reflect reasoned appraisal or an unsupported impression. Appropriate reliance concerns whether the clinician accepts or rejects AI appropriately, but a reliance decision can be correct by accident (31, 37). Verification is an observable act, but the act may be performed ritualistically without detecting anything. Calibration, reliance and verification therefore provide partial external expressions of alignment; none alone identifies the underlying case-bounded capacity to detect mismatch or error.

Measures that distinguish the basis of confidence rather than confidence magnitude alone may therefore be useful, although such approaches remain preliminary and, within BRACE, would need to remain item-level and case-bounded (95).

What must be detected varies with the reasoning process. Uncritical early convergence may trigger an optional prompt for an independent confidence, evidence-sufficiency or discriminator judgement before AI resolution is disclosed; rejection of a well-supported AI output should instead make the evidential conflict visible without presuming that the clinician is wrong. BRACE therefore supports metacognitive alignment by creating opportunities for detection and reappraisal rather than by deciding whether the clinician's reasoning is adequate.

Metacognitive alignment matters because it supports contingency preparedness: the capacity to reason safely when AI is unavailable or its output is contested or wrong. A clinician who cannot detect when an AI-supported diagnostic frame requires reconsideration cannot exercise that capability effectively. BRACE therefore seeks to keep detection, verification and reappraisal active during routine AI-supported interaction, rather than relying on their assessment only after AI support has been removed.

## 9 Governance of the protected developmental space

The inferential limits established in Section 5.3 require a governance architecture that makes formative use both credible and acceptable to clinicians. BRACE depends on clinicians being able to reason freely, without withholding uncertainty, doubt or provisional judgements because of concern about how the resulting record might later be used. Within the framework, uncertainty, hesitation, disagreement, revision, missed discriminators and incomplete reasoning pathways are treated as material for learning rather than evidence of individual failure (96, 97). The interactional and formative-review data from which such material is drawn therefore remain within a clinician-controlled protected developmental space.

Only two governed routes cross that boundary. First, with separate consent, de-identified findings from periodic review may contribute to longitudinal evaluation and system refinement only when reported in groups large enough to prevent identification of an individual clinician. Second, a clinician may initiate an anonymised expert review of possible AI error, unsafe output or missing contextual information. System configuration may receive only these aggregated, de-identified findings; it does not receive the clinician's developmental record itself. Individual learning needs are addressed within the protected space through the formative review described in Section 7.2 and do not constitute a further data route.

The longitudinal interactional record also remains within the protected developmental space. Patterns within that record, considered alongside AI-off formative reassessment, may inform the form and level of support appropriate for a particular clinician, within a particular case type, at a particular time. They are not intended to support broader judgements about the clinician's overall competence. BRACE therefore produces no competence score, comparative ranking or durable high-support label, and developmental data are unavailable to line management, appraisal, credentialing, employment or disciplinary systems.

These protections are necessary because the developmental value of the record depends on clinicians being able to reason freely while uncertainty remains unresolved. A provisional judgement spoken to a colleague is transient; once entered into a persistent digital record, the same uncertainty may be available to administrators, auditors or other parties whom the clinician did not envisage as its audience. Evidence from other settings suggests that the possibility of later access can alter what professionals are willing to record even when the underlying conversation remains open (98).

This matters particularly for BRACE because the material it seeks to preserve is precisely the material most vulnerable to self-censorship: provisional judgements, alternatives deferred rather than excluded, discriminators not pursued, unresolved uncertainty and points at which confidence exceeded the available evidence. An outcome can be documented retrospectively once uncertainty has disappeared; a reasoning record must be created while uncertainty is still present. A clinician who expects that such a record may later be used evaluatively may withhold or modify elements of that reasoning, removing the information on which formative adaptation depends. The protections are therefore not ethical decoration added to an otherwise functioning framework. They are a condition of the framework functioning as intended.

It follows that BRACE creates no escalation pathway from the developmental record into appraisal, supervision, credentialing or disciplinary processes. Concerns about professional competence remain within established systems of clinical governance, appraisal and supervision, with their own evidential standards, safeguards and rights of reply. Where patterns within BRACE suggest that additional learning support may be useful, the response remains formative and private through the review processes described in Section 7.2. Where an independent patient-safety incident occurs, ordinary clinical-governance processes may address it, but the developmental record is not converted into evidence for that process.

Operational controls are specified in Supplementary Material S1–S4. Adaptation must use a recent and locally justified window and remain inspectable, challengeable, suspendable and resettable by the clinician. Participation in developmental components is opt-in; consent to formative review and consent to contribute data to research or evaluation are separate and revocable. Declining developmental participation does not reduce case-level clinical support and is not disclosed to line management. A clinician who declines both forms of participation continues to receive case-level support but generates no longitudinal developmental record.

## 10 Limitations and evaluation agenda

### 10.1 Limitations and scope conditions

BRACE is a conceptual framework, and several of its central propositions require prospective testing. The interactional signals described in Section 5, the combinations used to trigger support, and the thresholds separating support configurations require validation for sensitivity, specificity, stability and acceptability. Implementation will also vary across clinical domains such as emergency medicine, oncology, radiology and primary care, where case structure, workflow, AI functionality and characteristic failure modes differ.

The present framework is deliberately centred on the clinician-AI dyad. Interprofessional and team reasoning are deferred rather than assumed to follow directly from the same architecture. A team-level extension is conceivable, but it would need to preserve discipline-specific reasoning, uncertainty and disagreement across handover while preventing the interactional record from becoming a mechanism for surveillance, hierarchy reinforcement or retrospective blame. Implications for pre-registration curricula lie outside the framework in the same way. Preparation for AI-supported practice that preserves and strengthens causal reasoning would equip future clinicians to interrogate AI recommendations, not merely to use them, where those recommendations conflict with underlying biomedical mechanisms.

A further limitation concerns the feasibility of the protected developmental space. BRACE assumes that interactional and formative data can remain separated from appraisal, credentialing and employment processes. Whether that separation can be maintained under real institutional conditions is uncertain. Data collected for one purpose are commonly extended to others over time through function creep (99). Implementation studies must therefore evaluate not only clinical and developmental outcomes, but whether BRACE remains bounded, formative and acceptable in routine practice.

The framework also draws substantially on evidence from neighbouring domains. Its mechanisms represent a principled extrapolation from allied areas of research, including human-automation interaction, cognitive psychology, learning and memory, rather than effects established directly in AI-supported clinical reasoning. The distinction is particularly important for capability preservation, where some of the nearest evidence comes from formal educational settings rather than longitudinal clinical practice. The evaluation programme below is intended to test rather than assume that these mechanisms generalise.

Questions of liability allocation between clinician, institution, developer and AI system are outside the present scope. They depend on jurisdiction-specific legal and regulatory arrangements that remain unsettled. BRACE instead adopts the accountability position developed in Section 4.2: clinical judgement remains with the clinician while the design of the interaction remains a system and institutional responsibility.

The consent architecture creates a further methodological limitation. Participation in formative review and contribution to research or evaluation are optional and separately consented. Clinicians who participate may differ systematically from those who decline, and low participation would constrain both implementation and the conclusions that could be drawn from longitudinal evaluation.

BRACE also assumes sufficient stability in the clinician-system relationship for recent interactional patterns to remain informative. Material changes to the underlying model, interface or support logic should trigger review and, where necessary, recalibration or resetting of affected case-type interaction histories, because previous patterns may no longer describe the same clinician-system relationship.

Finally, BRACE adds interaction to work that is already cognitively and temporally dense. Eliciting provisional alternatives, confirming verification, preserving a reasoning trace, offering post-case review and conducting periodic AI-off assessment all carry workflow costs. Pan et al. (60) identify workflow friction and alert burden as important threats to physician-facing AI safeguards. BRACE therefore distinguishes core requirements from developmental interventions that can vary in frequency. The inferential, adaptive and data-use bounds, the protected developmental space and the safety override are core requirements of the framework; developmental prompts and reviews may be applied selectively according to clinical context.

Because no framework can know in advance how much additional interaction can be accommodated within a given clinical setting, these limits must be established empirically, with a system implemented according to BRACE capturing most of the relevant measures directly. The interactional record already registers each developmental prompt, its timing and its outcome, so implementation studies can derive prompt frequency, additional interaction time, and rates of decline, override and abandonment directly from system use. Whether a clinician accepts or declines the optional offers described in Sections 5.3 and 6.3 is deliberately not recorded and therefore cannot influence subsequent adaptation. These measures describe the system's footprint, not the clinician; they are aggregated, de-identified and reported through the governed route that informs review of system configuration (Section 9). A system that protected reasoning by obstructing care would invert the framework's own purpose.

### 10.2 Evaluation agenda

BRACE makes two principal claims, and both are testable. The immediate claim is that bounded reciprocal support improves reasoning within an AI-supported encounter, reflected in outcomes such as detection of erroneous output, discriminator coverage and calibration relative to comparable output-centred support. The developmental claim is that preserving cognitive and metacognitive work across supported encounters protects subsequent independent capability, tested by whether the amount and type of work preserved predict later performance without AI.

Evaluation should therefore combine established human-AI outcomes with BRACE-specific developmental outcomes. Shared outcomes include detectability, correctability, non-criticality, automation bias and diagnostic robustness under load. BRACE-specific outcomes include adherence to the declared bounds, reasoning-state preservation across interruption and handover, discriminator coverage, rationale quality, metacognitive alignment, and preservation of AI-off capability. Patient-level safety and clinical outcomes remain necessary measures of implementation, but they cannot substitute for measures that directly test BRACE's proposed developmental mechanisms. Table 2 maps these principles to Tier 1 interactional measures and Tier 2 external anchor measures.

**Table 2:** Mapping BRACE design principles to measurable endpoints.

BRACE design principle	Corresponding measurable endpoint	Status of evidence
Tier 1. Intrinsic measures: generated by the clinician-AI interaction and requiring no separate assessment episode		
System uncertainty made visible (5.2)	Verification action rate following an uncertainty flag	New
Event-triggered verification checkpoints (5.1)	Automation-bias rate: omission and commission errors; negative consultation rate (correct independent judgement overturned by incorrect AI output)	Reused (37, 100)
Evidence-sufficiency prompting (5.1)	Confidence–evidence concordance across successive disclosure points	Partly reused (94)
Discriminator surfacing (5.1)	Discriminator coverage: proportion of relevant discriminators considered, by case type	New
Reasoning-state preservation (4.4)	Hypothesis continuity: retention of active hypotheses, deferred alternatives and reopening conditions across interruption, handover and re-presentation	Partly reused; extends existing handover instruments
Reciprocal adaptation (4.2, 4.4)	Cognitive- and metacognitive-work preservation by support configuration: proportion of encounters retaining independent generation, discrimination, verification and reflection at low, moderate and high support levels	New
Bounded inference (5.3)	Inference-drift audit: proportion of adaptations traceable to permitted signals; detection of adaptations that begin to rely on broader judgements about the clinician	New
Protected developmental space (9)	Integrity of the opt-in architecture and function of the expert review pathway under real employment conditions	New
Tier 2. Anchor measures: periodic, externally validated instruments, undertaken as formative review at the clinician's option, against which the intrinsic measures are calibrated		
Contingency preparedness (8.2)	AI-off independent diagnostic accuracy, benchmarked against the clinician's own prior baseline	Adapted; clinical post-withdrawal precedent and proposed longitudinal application (Budzyń et al., 2025; Ke et al., 2026)
Skill preservation: deskilling and never-skilling (8.1)	AI-off parallel-case performance related to the amount and type of cognitive **and metacognitive** work preserved across low-, moderate- and high-support encounters, conditional on baseline AI-off capability and case demand	Reused instrument, new longitudinal and clinician-specific application (75, 101)
Mis-skilling detection (8.1)	Reproduction of a known erroneous or biased representation on a later matched AI-off case following controlled exposure	New BRACE-specific endpoint
Metacognitive alignment (8.2)	Calibration curve; metacognitive sensitivity; treated as partial external expressions of alignment	Partly reused; no single measure identifies the underlying capacity (94)
Rationale quality as an indirect engagement proxy (5.1, 7.2)	Structured rating of reasoning documentation: interpretive summary, differential diagnosis, explanation of reasoning and alternatives considered; not treated as a direct measure of metacognition	Reused instrument, adapted interpretation (102)

Tier 1 measures arise from clinician-AI interaction; Tier 2 measures provide periodic external anchors. The final column indicates the extent to which each endpoint reuses, adapts or extends an existing measure, or is new to BRACE, with sources given where a measure is reused or adapted; Section 9 specifies data governance.

Evaluation must also establish that BRACE is being implemented as intended. Every adaptive action must remain traceable to a permitted signal and a case-level or case-type response. Any adaptation based on a prohibited person-level inference would therefore fall outside BRACE. This paper proposes the term inference drift for the gradual expansion from permitted case-bounded use of interactional data towards broader judgements about the clinician, a function creep specific to inference (Section 10.1). An inference-drift audit should therefore check whether the system's adaptations remain within the declared boundaries of BRACE. The audit evaluates the system, not clinician performance. Longitudinal within-clinician data are required because the framework concerns trajectories; static clinical datasets such as MIMIC-IV contain neither reasoning processes nor later unaided capability.

More demanding cases are likely to receive more support. Therefore, if greater support is associated with poorer later performance, this does not necessarily mean that the support caused the poorer performance; it may simply reflect that the clinician encountered more difficult, unfamiliar or uncertain cases. To separate the effect of BRACE support from the difficulty of the cases in which it is used, analyses should take into account the clinician's baseline AI-off capability for that type of case, together with case difficulty, novelty, evidence quality, system uncertainty and differences between clinicians.

What matters is not the level of support alone, but the amount and type of cognitive and metacognitive work preserved while that support is provided. The interactional record documents what support was provided and which generation, discrimination, verification and reflection operations were preserved. The developmental outcome is measured separately on matched or comparable cases subsequently undertaken without AI. Accuracy in the original supported encounter is not an adequate endpoint because an apparently successful outcome may arise from sound reasoning, effective AI assistance or fortunate error cancellation. Periodic AI-off anchors allow private recalibration of subsequent support while also providing the independent outcome against which cognitive- and metacognitive-work preservation can be evaluated.

Longitudinal association alone cannot establish which BRACE components cause an observed effect. Studies will therefore need to vary individual components prospectively at clinically safe decision points, including when AI output is shown, whether the clinician is first invited to state their own judgement, discriminator prompting and post-case review. Such variation must remain subordinate to the safety override specified in Section 4.4.

The three developmental risks require different tests. Deskilling and never-skilling can be examined through longitudinal AI-off performance, but distinguishing them requires the mechanism-specific predictions developed in Section 8.1. Script-level deskilling should produce increasing confusion between neighbouring presentations together with relatively rapid recovery after contrastive re-exposure; a script that never formed should show no equivalent savings. Mis-skilling requires controlled exposure to known erroneous or biased AI output, followed by later unaided assessment of whether the erroneous representation is reproduced.

The framework also makes predictions about specificity. Capability changes confined to case types in which critical cognitive and metacognitive work was weakly preserved would be consistent with the proposed schema-level mechanism, meaning that the effect is specific to the relevant clinical schema rather than to reasoning ability generally. This interpretation would be stronger if the relationship were demonstrated within the same clinicians rather than only between different clinicians. Decline across unrelated case types would instead imply a more general change in reasoning practice and would suggest that BRACE's case-bounded mechanism is insufficient to explain the change.

Most importantly, the central developmental claim is falsifiable. This test requires BRACE to be implemented as intended and differences in case difficulty and context to be taken into account. Under those conditions, the claim would not be supported if the amount and type of cognitive and metacognitive work preserved during supported encounters failed to predict subsequent AI-off capability. Support for the stronger claim that BRACE is protective requires evidence that interactions preserving more of the hypothesised cognitive and metacognitive work are followed by better maintained or improved independent capability.

Testing this programme will require longitudinal, within-clinician data across case types, settings and specialties. Collecting such data is likely to require multicentre collaboration.

## 11 Conclusion

This paper has proposed Bounded Reciprocal Adaptation for Clinician Engagement (BRACE), a framework that treats the clinician-AI interaction rather than the model output as the focus of design and evaluation. Its defining constraint is that AI may adapt the amount and form of support to the interaction surrounding reasoning within a specific case while inferring nothing about the clinician's general competence. These bounds preserve the candour required for the reasoning record to support development.

Three developmental risks threaten independent capability: script-level never-skilling, script-level deskilling and mis-skilling. Script-level never-skilling occurs when AI supplies the resolution and displaces the discriminating work through which an illness script would otherwise form. Script-level deskilling is the progressive weakening, through disuse, of access to a previously acquired illness script. Mis-skilling occurs when erroneous or biased AI output is incorporated into subsequent independent reasoning. All three may occur across a clinical career rather than being confined to training, and none can be inferred reliably from performance while AI support is present.

BRACE therefore makes a testable developmental claim: the amount and type of cognitive and metacognitive work preserved during an AI-supported encounter should predict the independent capability subsequently demonstrated without AI. The hypothesis fails if that relationship does not hold when BRACE is implemented as intended and differences in case difficulty and context are taken into account. The aim is a reciprocal partnership in which judgement and accountability remain with the clinician, who can still reason independently when AI is absent or its output is contested or wrong.
