# Supplementary material for: Rethinking AI in clinical decision support: a framework for reciprocal human-AI interaction
Source: Front Digit Health. 2026 Sep 11;8:1887161. doi: 10.3389/fdgth.2026.1887161 (PMC13612526; doi:10.3389/fdgth.2026.1887161)
Supplement: Supplementary file 1 [file Datasheet1.docx]

# Supplementary Material

## Rethinking AI in Clinical Decision Support: A Framework for Reciprocal Human-AI Interaction

Colin J. Greengrass*

*Correspondence: Colin J. Greengrass, cgreengrass@rcsi.com

This Supplementary Material sets out the governance provisions of the BRACE framework (S1–S4), two additional design configurations (S5–S6), and a note on ambient documentation systems (S7). The main text provides sufficient detail to assess BRACE’s conceptual claims, inferential boundaries, and proposed tests. The sections below provide additional operational detail, specify permitted configurations, and clarify the scope of those claims without introducing or modifying them. References cited here appear in the main reference list unless they are listed separately at the end of this document.

## Part A. Governance, Data Protection and Consent

### S1. Bounded Adaptation to Individual Interaction Patterns

The longitudinal interactional record described in Section 4.4 constitutes a limited form of user modelling. BRACE treats this modelling as provisional, bounded, and developmental. Its purpose is to calibrate support to the clinician, not to evaluate the clinician.

Patterns of interaction may provide information about how the clinician and system are working together. However, they must not be converted into claims about the clinician’s competence, motivation, professionalism, emotional state, fatigue, or stable personal characteristics. Nor should they be used to construct permanent clinician profiles.

BRACE therefore uses recent and current interactional patterns only to adjust the timing, framing, or depth of support. Any such adjustment must remain inspectable, challengeable, suspendable, and resettable by the clinician.

These requirements have specific operational meanings. **Inspectable** means that the clinician can see what adjustment is currently active and the interactional patterns on which it is based. **Challengeable** means that the clinician can contest an adjustment and that the challenge triggers review rather than simply being recorded. **Suspendable** means that the clinician can return the system to its default configuration for any period without providing a reason. **Resettable** means that accumulated interactional patterns can be cleared so that an atypical period of practice does not continue to influence future adaptation.

The minimum technical state necessary to implement these choices may be retained. However, whether or not a clinician inspects, challenges, suspends, or resets the system must not itself be treated as a behavioural signal or used to make any judgement about that clinician. Where the contextual configuration described in S5 is active, the “default configuration” refers to that contextual baseline. Suspending or resetting personalised adaptation therefore returns the system to the contextual baseline rather than disabling contextual safeguards altogether.

These restrictions are necessary because the same interactional data that can support learning and safer clinician–AI interaction could also be repurposed for performance management or surveillance. Data-protection authorities have identified this as an important risk of workplace technologies whose data-processing capabilities may exceed what is visible to those being observed (Article 29 Data Protection Working Party, 2017). Identifiable interactional data should therefore remain within a protected developmental space and be kept separate from summative judgement, employer surveillance, or disciplinary processes.

### S2. Engagement with Specific Regulatory Provisions

BRACE is designed to remain compatible with workplace AI and data-protection requirements. The relevant regulatory principle is narrow: interactional data generated during employment must not be repurposed for emotion inference, workforce monitoring, or clinician assessment.

This position is consistent with the EU AI Act, which places restrictions on workplace emotion recognition and treats certain systems used to monitor or evaluate workers as high-risk. Under the Act, emotion recognition is defined in relation to emotions or intentions inferred from biometric data (European Union, 2024, Article 3(39)). The definition does not include physical states such as fatigue; the Act specifically distinguishes systems that detect fatigue in professional pilots or drivers for accident-prevention purposes.

BRACE does not rely on that distinction as permission to infer fatigue. As stated in Section 5.3 of the main text, confidence ratings, overrides, verification behaviour, and formative-review traces remain developmental interaction signals. They must not be interpreted as proxies for clinician competence, psychological state, fatigue, or workforce performance.

The same distinction is relevant to the fatigue-related prompt described in Section 5.3 of the main text. The EU AI Act identifies as high-risk certain employment systems intended to monitor or evaluate the performance and behaviour of people in work-related relationships. BRACE is designed differently. Where a fatigue-related or heightened-support prompt is directed only to the individual clinician, is not shared with another party, and is not entered into an employment or performance record, its purpose is individual safety support rather than workforce monitoring.

The safeguards attached to such prompts are therefore architectural rather than merely procedural. The output must remain private to the clinician, must not enter an employer-accessible record, and must not be available for later performance evaluation. These design conditions support the intended regulatory boundary. However, the final legal classification of any deployed system will depend on its stated intended purpose, its actual use, and the law applicable in the relevant jurisdiction.

### S3. Data Governance

Interactional data generated by BRACE may include confidence ratings, override patterns, formative-review responses, schema-completion patterns, reasoning-tree interactions, and post-case review entries. These data are subject to non-negotiable governance requirements.

Processing must be restricted to three purposes: case-level diagnostic support, clinician development, and governed system calibration. Clinicians must be able to access their own data. Supervisors and employers must not have routine access to identifiable individual records. Any service-level metrics must be de-identified and sufficiently thresholded to reduce the risk of re-identification. Data retention periods must be explicitly defined and proportionate to their intended purpose.

Secondary use for performance management, productivity monitoring, disciplinary processes, or workforce planning is prohibited within the BRACE framework. This prohibition is not merely a default that may be overridden locally if another lawful basis for processing can be identified. The separation between developmental use and evaluative use is a constitutive feature of BRACE.

Research use is treated separately. As described in Section 7.1 of the main text, framework evaluation uses a separately consented dataset. The developmental record itself is not repurposed for research.

System calibration may nevertheless require structured expert review of recurrent or serious AI-output failures. If a clinician flags an output as unsafe, erroneous, insufficiently contextualised, inconsistent with local guidance, or potentially miscalibrated for a particular patient subgroup, the case should enter a protected review pathway. It should not trigger an automatic change in system behaviour.

Expert review should consider the case representation, the AI output, the system’s uncertainty state, the evidence used or omitted, and the relevant local clinical context. The purpose is collective calibration and diagnostic safety, not attribution of fault to the individual clinician. Any changes to thresholds, prompts, local configuration, or model behaviour should occur through a documented and auditable governance process.

### S4. Consent in the Employment Relationship

Participation in developmental components should be kept outside ordinary employer data flows so that a clinician’s decision not to participate is not visible to line management or interpreted negatively.

Consent is a particularly weak safeguard in employment settings because clinicians may feel that use of an AI-CDSS is expected as part of routine work. European data-protection guidance makes this point explicitly: because of the imbalance of power between employer and employee, workers are often not in a position to give, refuse, or withdraw consent freely, and consent is therefore seldom an appropriate legal basis for workplace processing (Article 29 Data Protection Working Party, 2017).

Within BRACE, opt-in status therefore governs participation in developmental components. It is not the legal or ethical basis for routine case-level decision support.

Tier 2 developmental functions, including confidence ratings, formative review, schema remediation, and uncertainty-rich vignette exercises, must therefore be optional, with no employment consequences for declining them. As set out in Sections 7.1 and 9 of the main text, BRACE distinguishes two independently revocable forms of consent: first, consent to participate in formative review; and second, consent for a pseudonymised copy of formative-review outputs to contribute to evaluation of the framework.

Declining either or both forms of consent leaves case-level decision support unaffected. However, functions that depend on longitudinal developmental storage or formative review will not be activated.

The clinician’s consent status should be stored only to the extent necessary to implement that choice. It must not become part of the developmental record, must not be visible to line management, and must not be used or interpreted for employment, workforce, or evaluative purposes.

## Part B. Further Configurations

### S5. Contextual Risk Parameters as Anticipatory Priors

A further configuration of BRACE would allow contextual risk parameters to act as anticipatory priors. In practical terms, this means that the system could adjust its baseline threshold for offering verification support before any clinician-specific interactional signal has appeared.

Examples include shift duration, time elapsed since the start of a shift, handover frequency, and known periods of high workload. These are institutional or environmental facts rather than psychological inferences about the clinician.

The relevant cognitive rationale is described in Section 5.3 of the main text. Using such contextual conditions to increase the sensitivity of verification prompts extends the minimal condition-triggered configuration described there. It also extends the signal restrictions described in Section 5.1 because the relevant inputs are contextual rather than interactional.

Importantly, the system would make no inference that an individual clinician is fatigued, impaired, or struggling. It would instead recognise that some working conditions are associated with a greater likelihood of the reasoning vulnerabilities described in Section 2.

For example, a clinician eight hours into a night shift after two handovers might receive an earlier verification checkpoint than a clinician two hours into a day shift with a stable case list. The reason is not that the system has detected difficulty in the first clinician. Rather, the surrounding conditions justify a more cautious baseline level of support.

This configuration would require strict governance. Contextual shift parameters could be used only to adjust support thresholds. They must not be stored as individual performance context, transferred into an employment record, or reused for workforce evaluation. They should be discarded when they are no longer required for the current shift and remain subject to the same principles of purpose limitation and data minimisation described in Section 9 (European Union, 2024).

### S6. Clinician-Controlled Support Elevation

A related configuration would allow clinicians to increase the level of support themselves.

Contextual parameters can establish a baseline level of support, but they cannot capture every circumstance that may affect a particular shift. The clinician will often know things about the situation that the system cannot know. BRACE would therefore allow the clinician to choose, for the current shift, to operate with more sensitive verification prompts, more prominent uncertainty displays, and stronger preservation of the reasoning state.

A minimal additional feature could allow clinicians to view their own decision latency during the current shift. This information would be displayed descriptively rather than interpreted by the system and would disappear from the clinician-facing display at the end of the shift. Any interpretation of the pattern would therefore remain the clinician’s own. The underlying latency data, where retained as part of the developmental record, would remain subject to the protections set out in Section 9.

The governance of this control is more important than the mere existence of the control. The system should not ask why the clinician has requested additional support, and no reason should be recorded or inferred. The request should exist only as a temporary control state for the current shift, should be visible to no one else, and should be deleted when the shift ends.

Similarly, neither use nor non-use of the control should be available for aggregation, review, or interpretation. This follows directly from the distinction between transient states and stable traits discussed in Section 5.3. If the system recorded why a clinician requested extra support, or accumulated such requests across shifts, the record could become a proxy for perceived vulnerability. BRACE therefore avoids making that inference altogether: the clinician simply chooses the level of support.

The same principle applies to preferences set during baseline calibration. A clinician may specify that they want support sensitivity to increase earlier in a shift than the contextual default would provide and may revise that preference at any time. They may not, however, reduce support below the contextual baseline. This asymmetry is deliberate and follows from the safety rationale described in Section 5.3 of the main text.

Aviation provides a limited precedent for the underlying principle. Regulation distinguishes fatigue-related safety notification to an individual operator from workplace emotion inference (European Union, 2024). The methods used in BRACE are, however, materially different. BRACE uses no physiological, biometric, or ambient-sensed input, consistent with Section 5.4. Its contextual baseline is derived from institutional facts about the shift rather than from measurement of the clinician. The aim is therefore to preserve the protective function while avoiding the surveillance infrastructure that may accompany direct physiological monitoring.

### S7. Ambient Documentation Systems

Systems that capture clinical encounters and use them to generate documentation are increasingly deployed, but they raise governance questions that BRACE does not attempt to resolve.

Their governance differs from that of the framework in at least three important respects.

First, ambient documentation primarily raises questions of patient consent rather than clinician consent, together with issues of secondary data use and governance that are specific to continuous or ambient capture in clinical settings (Martinez-Martin et al., 2021).

Second, many ambient documentation systems fall outside medical device regulation because they are intended to document rather than diagnose or recommend. They may therefore be classified as administrative tools rather than medical devices, leaving some questions about safety, efficacy, and oversight less clearly addressed despite the sensitivity of the data they process (Topaz et al., 2025).

Third, the lawfulness of recording depends on jurisdiction-specific rules governing audio capture and eavesdropping. These rules differ in whether one-party or all-party consent is required, and their application to ambient clinical recording is not yet fully settled and remains the subject of legal dispute (Anderson et al., 2026).

Whether records generated by ambient documentation systems could appropriately serve as inputs to BRACE is therefore left unresolved pending greater regulatory, legal, and governance clarity.

## Supplementary References

Anderson, T. N., Sinha, M. S., Cohen, I. G., & Jones, R. L. (2026). Ambient AI in clinical practice: The legal landscape of recording consent requirements. *NEJM AI, 3*(6). https://doi.org/10.1056/AIp2600203

Article 29 Data Protection Working Party. (2017). *Opinion 2/2017 on data processing at work (WP249).* European Commission.

Martinez-Martin, N., Luo, Z., Kaushal, A., Adeli, E., Haque, A., Kelly, S. S., Wieten, S., Cho, M. K., Magnus, D., Fei-Fei, L., Schulman, K., & Milstein, A. (2021). Ethical issues in using ambient intelligence in health-care settings. *The Lancet Digital Health, 3*(2), e115–e123. https://doi.org/10.1016/S2589-7500(20)30275-2

Regulation (EU) 2024/1689 of the European Parliament and of the Council of 13 June 2024 laying down harmonised rules on artificial intelligence (Artificial Intelligence Act), OJ L, 2024/1689.

Topaz, M., Peltonen, L. M., & Zhang, Z. (2025). Beyond human ears: Navigating the uncharted risks of AI scribes in clinical practice. *npj Digital Medicine, 8*, 569. https://doi.org/10.1038/s41746-025-01895-6
